# Crystallization and Dielectric Properties of MWCNT /Poly(1-Butene) Composite Films by a Solution Casting Method

**DOI:** 10.3390/ma13030755

**Published:** 2020-02-07

**Authors:** Lingfei Li, Qiu Sun, Xiangqun Chen, Yongjun Xu, Zhaohua Jiang

**Affiliations:** 1 School of Chemistry and Chemical Engineering, Harbin Institute of Technology, Harbin 150001, China; 2 School of Materials Science and Engineering, Harbin Institute of Technology, Harbin 150001, China

**Keywords:** poly(1-butene), multi-walled carbon nanotubes, solution crystallization, dielectric properties, breakdown strength

## Abstract

In this work, poly(1-butene) (PB-1) composite films with multi-walled carbon nanotubes (MWCNT) were prepared by a solution casting method. The relationship between the dielectric properties and the crystal transformation process of the films was investigated. It was indicated that there were two crystal forms of I and II of PB-1 during the solution crystallization process. With the prolongation of the phase transition time, form II was converted into form I. The addition of the conductive filler (MWCNT) accelerated the rate of phase transformation and changed the nucleation mode of PB-1. The presence of crystal form I in the system increased the breakdown strength and the dielectric constant of the films and reduced the dielectric loss, with better stability. In addition, the dielectric constant and the dielectric loss of the MWCNT/PB-1 composite films increased with the addition of MWCNT, due to the interfacial polarization between MWCNT and PB-1 matrix. When the mass fraction of the MWCNT was 1.0%, the composite film had a dielectric constant of 43.9 at 25 °C and 10^3^ Hz, which was 20 times that of the original film.

## 1. Introduction

Recently, energy storage film capacitors have become a focus for all over the world, due to the increasing depletion of fossil fuels and the growing environmental issues [1,2]. Polymer-based dielectric materials have unique advantages for use as diaphragms, an important part of capacitors [3,4,5]. For example, biaxially-oriented polypropylene (BOPP) films (ε’ = 2.2, U_d_ < 2 J·cm^−3^) are commonly used in capacitors. However, the working temperature of BOPP is limited to 85 °C, which will significantly shorten its life at high temperatures [6,7,8]. In comparison with a BOPP film, poly (1-butene) (PB-1) has a dielectric constant of 2.53 and a dielectric loss of 0.0005 at 10^3^ Hz [9]. What is more, it has better mechanical properties, chemical stability, and good creep resistance at high temperatures (100 °C) [10], which can be used as a diaphragm for a storage capacitor.

There are five kinds of crystal forms in PB-1, which is a semi-crystalline polymer: I, II, III, I’, and II’’ [11]. Thus, crystal transformation exists in the actual processing. It has been reported that the unstable crystal form II is first formed in the melt solidification process, and the mechanical properties are poor [12]. Despite this, crystal form II can spontaneously change into a stable crystal form I during the room temperature placement [13], leading to the improved mechanical properties. However, the transformation process, which took several weeks, could led to volume shrinkage. It has been noted that the transition process from crystal II to crystal I could be accelerated by introducing multi-walled carbon nanotubes (MWCNT) [14], glycidyl methacrylate [15], silicate [16,17], and other fillers into the PB-1 during the melt blending. However, crystal transformation caused by adding filler during the solution crystallization of PB-1 has rarely been reported.

Though the PB-1 matrix has high breakdown field strength and low dielectric loss, its dielectric constant is too small for the practical application. Today, conductive fillers are usually added to improve the dielectric performance of PB-1. For example, Ping et al. [18] prepared reduced graphene oxide/PB-1 composite films, in which graphene nanosheets not only improved the nucleation ability, crystallinity, and thermal stability of the film, but also increased the storage modulus of the film. It was found that the dielectric constant of the composite films increased with increasing graphene nanosheets. At 10^3^ Hz, when the mass fraction of filler was 2%, the dielectric constant of the film increased to 33.9, while its dielectric loss reached 1.5, which was useless for the practical application. Wanjale and Jog [19] prepared MWCNT/PB-1-based composites by melt hot pressing, and discussed their viscoelastic and dielectric properties. The study indicated that in the elastic region, the storage modulus increased significantly, and the relaxation behavior of the molecular chain was delayed due to the presence of MWCNT. When the mass fraction of MWCNT was 7%, the dielectric constant and conductivity of the composites were significantly improved, the dielectric constant increased from 2.2 to 70, and the electrical conductivity increased significantly from 10^−15^ to 10^−3^ S/cm. All the reported conductive fillers/PB-1 matrix composite films were prepared by melt blending. Though the dielectric constant can be increased by adding conductive fillers, the high content of fillers will led to large dielectric losses, which will increase the leakage current and the capacitor will be more easily broken down.

In addition, PB-1 is a linear polymer, and the energy storage density (U_d_) can be formulated as U_d_ = 1/2ε’ε_0_E_b_^2^ [20,21], where ε_0_ represents the vacuum dielectric constant 8.854 × 10^−12^ F·m^−1^. The good energy storage density is not only related to the high dielectric constant (ε’), but also depends on high breakdown strength (E_b_). To our knowledge, a discussion of the effects of MWCNT on the breakdown strength of a PB-1 composite film has not been reported.

In this paper, PB-1 was used as the matrix material and MWCNT were used as the filler. The PB-1 film and MWCNT/PB-1 composite film were prepared using a solution casting method, due to its simple process, low filler adding content, and good reproducibility. The microstructure of the films was characterized by X-ray diffraction (XRD), infrared spectroscopy (IR), and differential scanning calorimetry (DSC). The crystal transformation caused by adding MWCNT during the solution casting was analyzed. The effects of crystal form and the amount of MWCNT on the dielectric constant, dielectric loss, and breakdown characteristics of the PB-1 composite films were investigated.

## 2. Materials and Methods

### 2.1. Materials

The PB-1 was purchased by Lyondell Basell Industries, Hoofddorp, Holland, under the trade name PB 0110M, with a melt index of 1.333 g/10 min (230 °C, 2.16 kg) and average of molecular weight M_w_ = 711 kg/mol. MWCNT with the average diameter of 20−40 nm and length of 5 μm were purchased from Shenzhen Nanotech Port Co, Ltd. (Shenzhen, China) and purified before delivery (amorphous carbon mass fraction less than 3%). Decalin was used as the solvent and was provided by Aladdin Reagent (Shanghai, China).

### 2.2. Preparation

**Figure 1:**
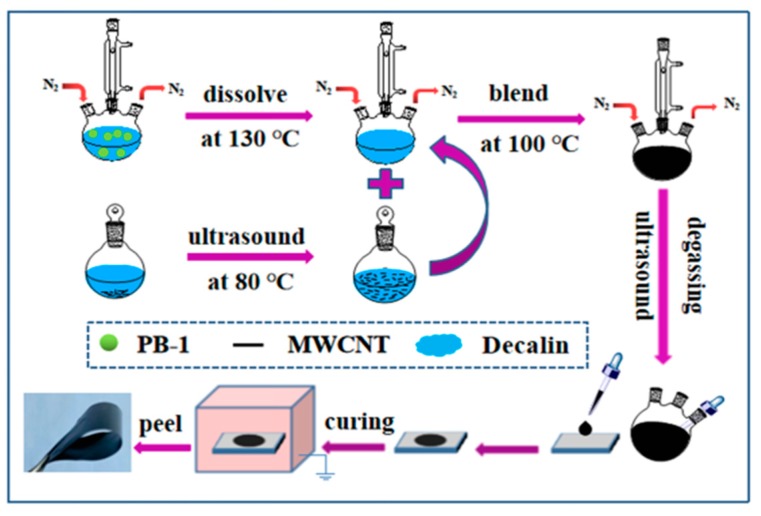
Schematic representations showing the fabrication of the MWCNT-modified PB-1 composite films.

Firstly, PB-1 resin was quickly dissolved in decalin solvent at 130 °C to prepare 8% (mass percent) of the PB-1 solution under nitrogen atmosphere. MWCNT were dispersed in the decalin at 80 °C by ultrasonication (ultrasonic cleaner BL10-250B, 59 kHz, 250 W sonic power, Bilon instrument manufacturing co, Ltd. Shanghai. China), and this system was kept at 80 °C for 1 h. The MWCNT colloidal dispersion and the PB-1 polymer solution were mixed and stirred for 3 h with sufficient agitation at 100 °C. Then, the polymer mixed solution was ultrasonicated at 80 °C for 1 h. After static defoaming, it was poured onto a glass plate and extended to obtain a uniform initial film. Next, the films were annealed at 100 °C for 24 h in a drying oven. Then, the film was moved to a vacuum oven at 100 °C for 24 h to remove the solvent.The pure PB-1 films without added MWCNT were marked as PB-X h (X represented phase transition time). And the MWCNT/PB-1 composite films were marked as PBC ω%-X h (where PB represents PB-1, C represents MWCNT, and ω% represents the mass fraction of the MWCNT in the PB-1). The thickness of the prepared films was between 60–70 μm. The preparation process of the MWCNT/PB-1 composite films is shown in Figure 1.

### 2.3. Characterization

X-ray diffraction (XRD) measurements were performed using a X’Pert Pro X-ray diffractometer (PANalytical B.V., Almelo, Holland) with CuKα radiation (λ = 0.154 nm) with a scanning speed of 10°/min from 7° to 90°. Fourier-transform infrared spectroscopy (FT-IR) was performed with a Bruker VERTEX 80 spectrometer (Bruker, Karlsruhe, Germany) over a range of 650–4500 cm^−1^. The sample was cryogenically fractured in liquid nitrogen, and the fractured surface of films was sprayed with gold. Scanning electron microscope (SEM) (S4800, Japan Hitachi, Tokyo, Japan) was used to observe the fracture surface morphology at an acceleration voltage of 20 kV and a magnification of 7000. The thermal analyses of the polymer were performed with a differential scanning calorimetry instrument (type DSC-Diamond, Perkin-Elmer, Waltham, USA). The heating and cooling rates were 10 °C/min. The degree of crystallinity (X_C_) of the specimen could be calculated according to following formula:

X_C_ = ΔH_m_ / ω ΔH^0^_m_ × 100% (1)

where ΔH_m_ was the melting heat of the composite films, ω was the weight percentage of PB-1, and ΔH^0^_m_ was the melting enthalpy of PB-1 of 100% crystallinity, which was set to 125.4 J/g [22].

The PB-1 pure film and the MWCNT/PB-1 composite film were subjected to Thermogravimetric analysis (TGA). The thermal analyzer (model Perkin Elmer, Waltham, USA) was raised from room temperature to 600 °C under an air atmosphere, and the heating rate was 10 °C/min.

A broadband dielectric spectrometer (model Novocontrol GmbH Concept 40, Karlsruhe, Germany) was used to determine the dielectric properties and conductivity of films under room temperature (25 °C) over a frequency range of 100 to 1.0 × 10^7^ Hz.

A breakdown voltage tester (model TH9102B, Tonghui Electronics, Changzhou, China) was used to measure the dielectric breakdown strength of the sample. The prepared composite film and pure film materials were tested by direct current (DC) voltage breakdown test. The electrode and samples were soaked in cable oil, and the voltage was uniformly increased at a rate of 1 kV/s. The breakdown voltage U was recorded, the thickness of the breakdown point d was measured, and the breakdown field intensity E was calculated by Weibull distribution function.

## 3. Results and Discussion

### 3.1. Effect of MWCNT on Phase Transformation of PB-1 Thin Films

#### 3.1.1. Effect of Phase Transition Time on the Crystal Form Transformation of Pure PB-1 Films

**Figure 2:**
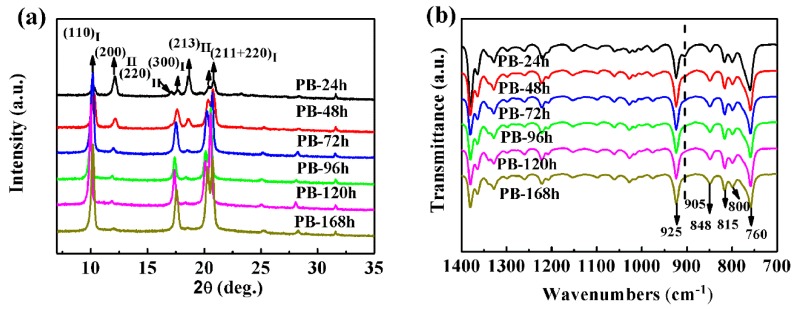
(**a**) XRD profiles and (**b**) FT-IR spectra of the PB-1 with different phase transition times.

The crystallization of the PB-1 solution could generally generate forms III and I’ [23], and the crystal structures were mainly affected by the temperature, solvent, and additives. When the PB-1 film was used in capacitors, it was desirable to obtain a stable crystal form I. Therefore, PB-1 films annealing at 100 °C were selected, avoiding formation of forms III and I’ [24]. The type of PB-1 film crystal form was observed by XRD and FT-IR, as shown in Figure 2, and the phase transition time was changed to obtain a series of PB-1 film samples.

As shown in Figure 2a, the diffraction peaks of PB-1 crystal form II, corresponding to crystal faces of (200), (220), and (213), were located at 2θ = 11.9°, 16.9° and 18.5°, respectively [25]. The characteristic diffraction peaks of form I were at 2θ = 10.0°, 17.5°, and 20.4°, corresponding to (110), (300), and (211 + 220) crystal faces [26]. It was observed that two crystal forms, forms I and II, were mainly present during the crystallization of the solution, due to the high annealing temperature. And as the phase transition time was prolonged, the intensity of the characteristic diffraction peaks of form II gradually decreased, while that characteristic diffraction peak of form I gradually increased. The conversion of form II to form I was illustrated.

As shown in Figure 2b, with the phase transition time prolonged, the intensity of the absorption peak of 905 cm^−1^ gradually decreased, and those of 925 cm^−1^, 848 cm^−1^, and 815 cm^−1^ were slightly enhanced. It has been reported that 905 cm^−1^ was the characteristic absorption peak of crystal II, and 925 cm^−1^ was the characteristic absorption peak of crystal I [27]. As a result, it was confirmed that in the initial stage of solution crystallization, forms I and II were present, and the conversion of form II to form I occurred.

#### 3.1.2. Effect of Phase Transition Time on Phase Transformation of MWCNT/PB-1 Composite Film

**Figure 3:**
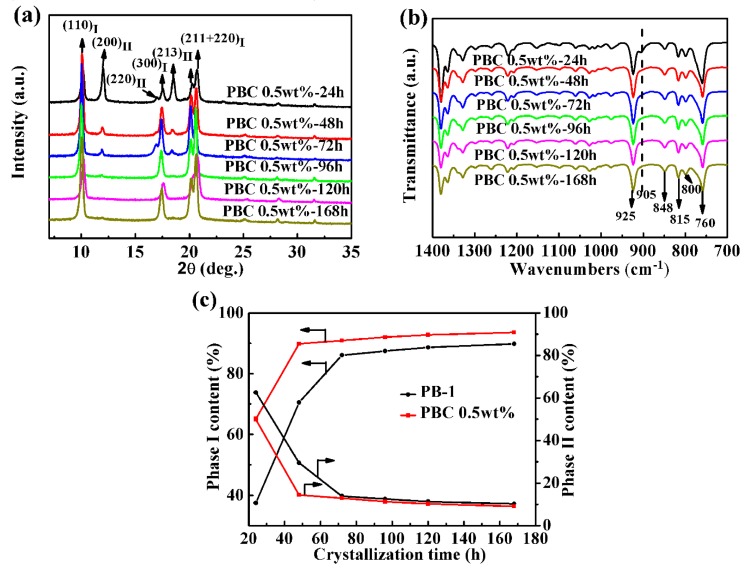
(**a**) XRD profiles and (**b**) FT-IR spectra of MWCNT-modified PB-1 composite films with different phase transition times, (**c**) contents of form I and II with different phase transition times at room temperature.

In order to apply the PB-1 film better, it was key to study how to shorten its phase transition period. MWCNT, as an ideal filler, could improve the mechanical properties, electrical properties, and thermal stability of polymers [28]. In this paper, the crystallization properties of PB-1 composite films with MWCNT of 0.5 wt% were investigated by XRD and FT-IR, with alterations of the phase transition time, as shown in Figure 3.

It could be seen from Figure 3a that the PB-1 composite film modified by MWCNT still had two crystal forms, I and II. During the crystallization process, with the phase transition time prolonged, the intensity of the characteristic diffraction peaks of crystal form II decreased rapidly, and those of crystal form I were clearly enhanced. From the FT-IR results given in Figure 3b, it could be seen that the content of crystal form II was significantly reduced after crystallization for 48 h, compared to the original PB-1 film sample.

In order to indicate the speed of the phase transition from form II to form I in PB-1, the relative content of each crystal form was calculated by the area ratio of the characteristic diffraction peaks of form I and form II at 2θ = 10.0° and 2θ = 11.9°. The formulas were as follows, K_I_ = H_10.0_/(H_10.0_ + H_11.9_) and K_II_ = H_11.9_/(H_10.0_ + H_11.9_). The calculation results can be seen in Figure 3c. From the calculation results in Figure 3c, crystal form I’s content in the pure PB-1 film was 37.5% after crystallization for 24 h, while crystal form I’s content in the MWCNT composite film was 50.2%. When the phase transition time was further extended to 48 h, the content of form I in the modified PB-1 composite film was increased by 15%, compared to the pure PB-1 film. It was indicated that the addition of a small amount of MWCNT accelerated the conversion of form II to form I, and MWCNT played the role of heterogeneous nucleation during solution crystallization. With the prolongation of phase transition time, the content of form I in the modified PB-1 composite film was significantly higher than that of the original sample, and the transformation period of form II to form I was effectively shortened during solution crystallization. Thus, it became easier to obtain a PB-1 film with a stable crystal form I that could be used in industry and in commerce.

#### 3.1.3. Effect of the MWCNT Contents on the Crystallization Properties

##### XRD Results

**Figure 4:**
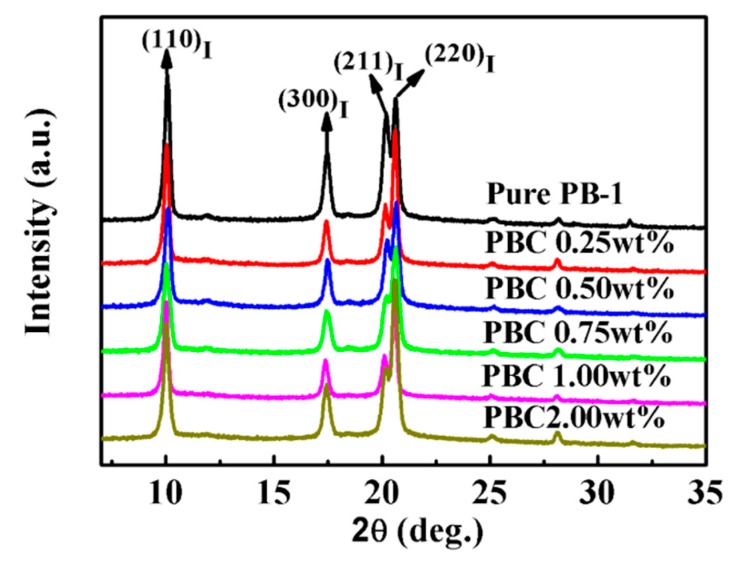
XRD patterns of pure PB-1 film and its composite films with different MWCNT contents.

In order to further study the effect of MWCNT contents on the crystallization properties of MWCNT/PB-1 composite films, the contents of MWCNT ranged from 0.25 wt% to 2.0 wt%, and the composite film samples with a phase transition time of 240 h were characterized by XRD, as shown in Figure 4.

It can be seen from Figure 4 that in the pure PB-1 film and the MWCNT/PB-1 composite film samples, the peaks at 2θ = 10.0°, 17.5°, 20.2°, and 20.6° corresponded to (110), (300), (211), and (220) crystal faces of form I. After the addition of MWCNT, no new diffraction peaks appeared, indicating that MWCNT were uniformly distributed in the PB-1 matrix. As the phase transition time was further extended to 240 h, almost all of form II in the system was converted to form I.

##### SEM Results

**Figure 5:**
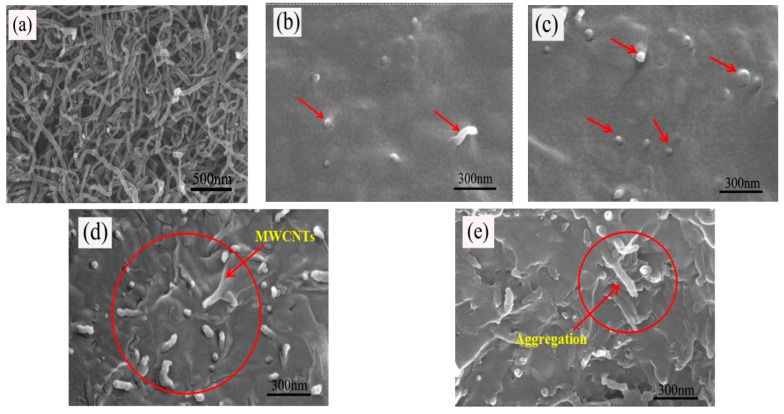
SEM micrographs of MWCNT original sample (**a**) and of the fracture surfaces of the composite films, wherein the content of the MWCNT is (**b**) 0.25 wt%; (**c**) 0.50 wt%; (**d**) 1.0 wt%; and (**e**) 2.0 wt%.

The SEM micrographs of the MWCNT powder sample and the fracture surface of MWCNT/PB-1 composite films with MWCNT ranged from 0.25 wt% to 2.0 wt%, and are shown in Figure 5.

It can be observed from Figure 5a that the diameter of the MWCNT was about 40 nm and the MWCNT were in an aggregated state. The bright spots and the tubular bright lines indicated by the arrows in Figure 5b–e are carbon nanotubes. The cavitation effect of ultrasound could generate a great force when the cavitation bubbles collapse. This force could overcome the van der Waals force between the MWCNT, which would contribute to the fragmentation and disentanglement of MWCNT, so that MWCNT could be more uniformly dispersed in the polymer matrix [29,30]. MWCNT were tightly bound around the PB-1 resin when the MWCNT contents were 0.25 wt% and 0.50 wt%. As shown in Figure 5b,c, the MWCNT could be more uniformly embedded in the PB-1 matrix with good compatibility. There was a certain distance between the carbon nanotube particles without connection. As seen in Figure 5d,e, it was difficult to disperse a higher content of MWCNT completely and evenly throughout the host polymer. When the content of the MWCNT was 2.0 wt%, local clusters of particles formed a micro-capacitor structure, as shown in Figure 5e. This three-dimensional conductive structure could transform the film from an insulator to a semiconductor.

##### DSC Results

**Figure 6:**
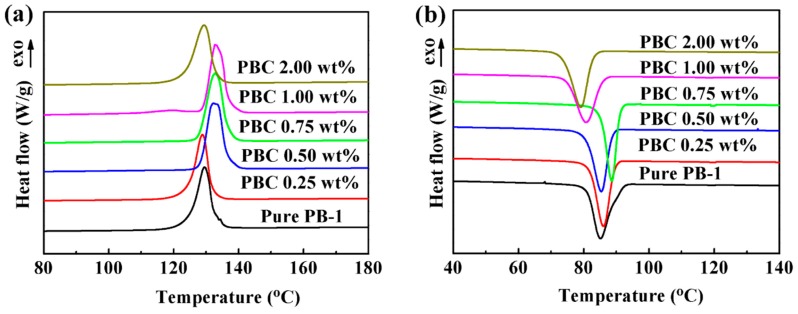
DSC (**a**) heating and (**b**) cooling curves of pure PB-1 and its composite films with different MWCNT contents.

After the PB-1 film was modified by MWCNT, the crystallization characteristics were changed to some extent. The DSC curves of the modified PB-1 composite films with different addition amounts of MWCNT after 240 h of crystallization are shown in Figure 6, and the corresponding crystallinities are listed in Table 1.

Figure 6a,b show the melting and crystallization curves of different contents of MWCNT/PB-1 composite films. The corresponding crystallization parameters are summarized in Table 1. As shown in Figure 6a,b and Table 1, it was indicated that the melting peak temperature and crystallization peak temperature of the MWCNT/PB-1 composite film were significantly improved in comparison to that of the pure PB-1 film. The characteristic curve of form I further indicated that form II was completely converted to form I. When the content of the MWCNT was 0.75 wt%, the crystallization peak temperature of the PB-1 film was increased from 85.11 °C to 88.57 °C, indicating that the presence of MWCNT enhanced the crystallinity of the PB-1 film by heterogeneous nucleation [31].

**Figure 7:**
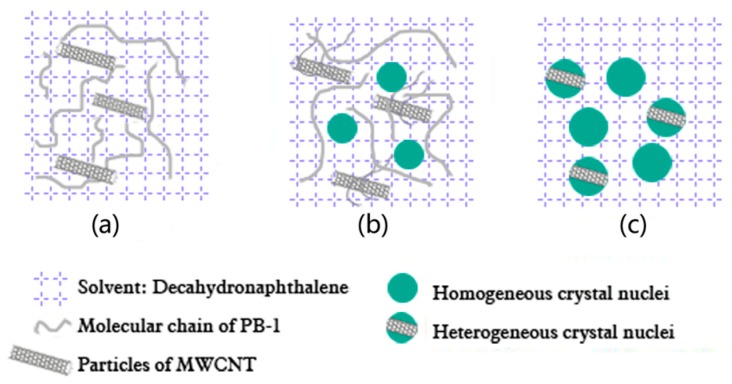
Proposed model of the solution crystallization of MWCNT-modified PB-1 composite films.

In order to illustrate the solution crystallization process of PBC more clearly, a crystallization model of PBC in a solution system was proposed, as shown in Figure 7. As a kind of nano-filler, the MWCNT had a heterogeneous nucleation effect on PB-1 during the crystallization of PB-1. When the content of MWCNT was less than 1.0 wt%, it increased the crystallization rate and crystallinity. Moreover, the crystallization nucleation rate was increased, and the PB-1 crystallization cycle was shortened. However, when the content of the MWCNT reached 2.0 wt%, the crystallization and melting peak temperature of the composite film began to decrease. The crystallization peaks of the composite films gradually broadened and the crystallization enthalpy decreased, indicating that the addition of an appropriate amount of MWCNT was more conducive to the ordered arrangement of PB-1 molecular chains during the crystal growth process. With the addition of a large number of MWCNT, local clusters of particles formed a micro-capacitor structure (as shown in Figure 5e), the activity space of the PB-1 molecular chain became smaller, and the activity of the molecular chain was weakened. Thus, the final crystallization enthalpy was lowered, and the crystallinity was decreased.

**Figure 8:**
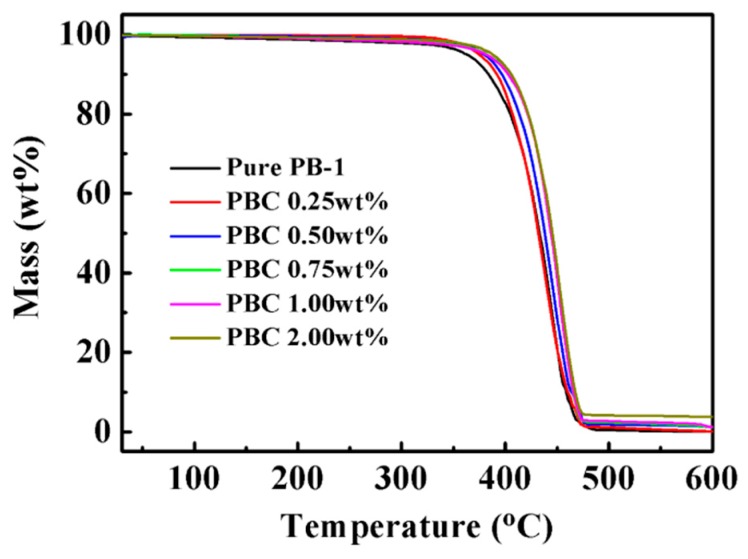
TGA curves of pure PB-1 and its composite films with different MWCNT contents.

In order to study the thermal stability of MWCNT/PB-1 composite films, the TGA of pure PB-1 film and MWCNT/PB-1 composite films are shown in Figure 8.

From the results shown in Figure 8, the thermal degradation curves for both the pure PB-1 film and MWCNT/ PB-1 composite films were typical one-step degradation behaviors. The pure PB-1 membrane began to decompose at 363 °C (temperature for 5% weight loss), and the main chain pyrolysis decomposed rapidly at about 475 °C with the largest weight loss. Pure PB-1 experienced almost full pyrolysis. However, with the increased content of MWCNT, the decomposition temperature rose to 387 °C. When the mass percentage of MWCNT was 2.0% (PBC-2.0 wt%), the MWCNT had a good blocking effect on the PB-1 matrix, which could enhance the thermal stability of the matrix. Furthermore, it was found that the carbon content of MWCNT/PB-1 composite films increased with the increase of MWCNT filler content, contributing to the carbonization of MWCNT in the PB-1 matrix [32].

### 3.2. Influence of the Introduction of MWCNT on the Dielectric Properties of PB-1 Films

#### 3.2.1. Effect of Phase Transition Time on Dielectric Properties

**Figure 9:**
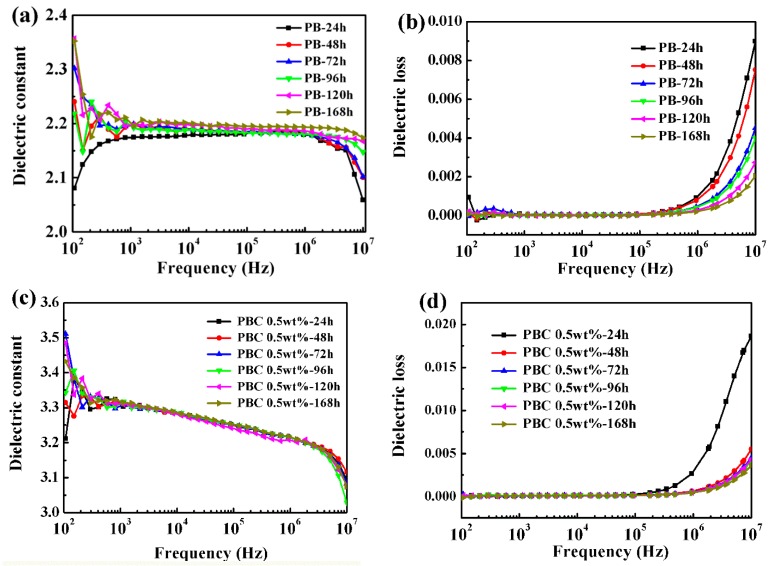
Dielectric constant and dielectric loss of PB-1 films (**a**,**b**) and MWCNT/PB-1 composites films (**c**,**d**).

In order to discuss the relationship between phase transformation and dielectric properties of PB-1 films, the dielectric properties of PB-1 films and MWCNT-modified PB-1 composite films were tested at room temperature, as shown in Figure 9.

Figure 9a,c showed the dielectric constant (ε’) of pure PB-1 and MWCNT/PB-1 composite films with different phase transition times, changing with frequency. The results indicated that the ε’ of the pure PB-1 and MWCNT/PB-1 composite films increased slightly with the phase transition time at the test frequency, due to the presence of a large amount of crystal form I in the system. After 168 h of phase transition, the ε’ of the pure PB-1 film reached 2.20. At low frequencies of 10^2^–10^5^ Hz, the ε’ of the PB-1 film changed slightly with the frequency. At high frequencies (more than 10^5^ Hz), the ε’ of the film decreased, due to the relaxation of the material itself. It was also found that the ε’ of the PB-1 composite film was increased by 1.5 times at a frequency of 10^3^ Hz after adding 0.5 wt% of MWCNT. Moreover, the addition of MWCNT did not change the relaxation phenomenon of the polymer itself, and the ε’ was still lowered at high frequencies.

It could be seen from Figure 9b,d that the dielectric loss (tan δ) of the pure PB-1 and MWCNT/PB-1 composite films varied with different phase transition times and the testing frequency. It was found that the tan δ of the pure PB-1 films and MWCNT/PB-1 composite films hardly changed with the frequency in the low frequency range of 10^2^–10^5^ Hz. However, the tan δ gradually decreased with the phase transition time when the testing frequency was more than 10^5^ Hz, which was also related to the decrease of the unstable crystal form II content. The results also showed that the addition of MWCNT caused a slight increase in the tan δ of the MWCNT/PB-1 composite film, and the polarization loss increased due to the interfacial polarization between the MWCNT and the PB-1 matrix.

#### 3.2.2. Effect of Phase Transition Time on Breakdown Strength

**Figure 10:**
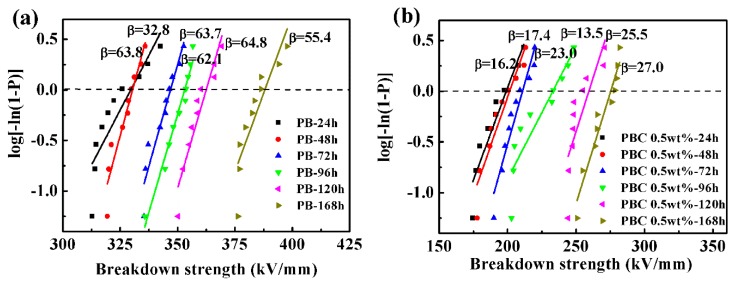
Dielectric breakdown strength of PB-1 films (**a**) and MWCNT/PB-1 composites films (**b**).

The breakdown field strength is also an important indicator for the energy storage density [33]. The variation of phase transition time, as well as breakdown strength of the pure PB-1 films and MWCNT/PB-1 composite films, are shown in Figure 10.

The calculation results of the breakdown strength using the Weibull distribution method are shown in Figure 10a,b, the equation is as follows [34,35]:

(2) $$P=1-\exp[{-(E/E}_{0}{)}^{\beta }]$$

Where P is the cumulative probability of electric failure, E is the test experimental breakdown strength, and E_0_ is the breakdown strength of the composite films at a cumulative breakdown probability of 63.2%, and β is a shape para, which indicates the degree of dispersion of experimental results. The PB-1 film and MWCNT/PB-1 composite film samples had a significant increase in breakdown strength with the phase transition time. It indicated that the existence of a large amount of stable crystal form I could promote the breakdown strength. The pure PB-1 film had a higher β value, and the film had better reliability and fewer defects. After the addition of MWCNT, the composite films with crystallization for 168 h produced defects due to the accumulation of nanoparticles, resulting in a change of breakdown field strength from 388 kV/mm to 276 kV/mm.

### 3.3. Determination of Percolation Threshold

#### 3.3.1. Conductivity of Modified PB-1 Films with Different Contents of MWCNT

**Figure 11:**
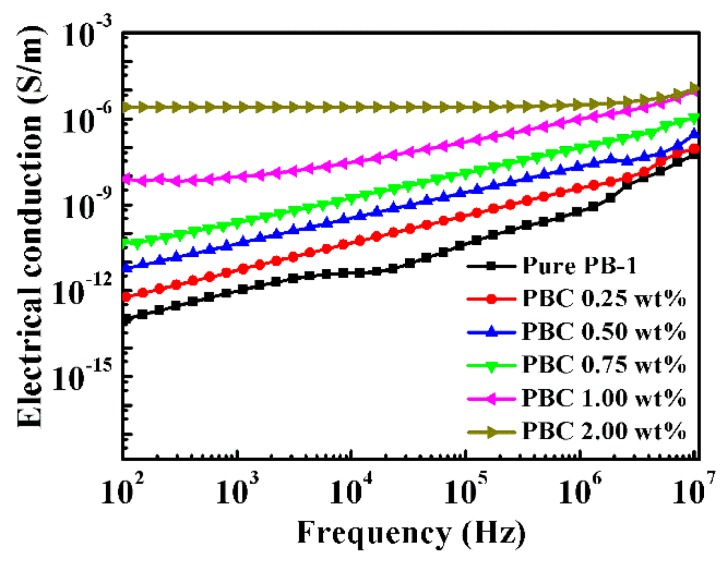
Frequency dependence of the conductivity of pure PB-1 and its composite films with different MWCNT contents.

Electrical transport in polymer-based composite films can also occur either through direct contact between conductive fillers or tunneling electrons between sufficient close conductive particles. Figure 11 shows the frequency dependence of the conductivity of MWCNT/PB-1 composite films with different filler contents.

It can be seen from the results of Figure 11 that when the content of MWCNT was less than 1.0 wt%, the electrical conductivity of MWCNT/PB-1 composite film increased slightly. This was mainly due to the uniform dispersion of MWCNT in the matrix without forming a conductive network, and the tunneling mechanism accounted for the dominant position [36]. When the conductive filler content increased from 1.0 wt% to 2.0 wt%, the electrical conductivity of MWCNT/PB-1 composite film increased from 10^−8^ S/m to 10^−6^ S/m, because the system formed a large number of micro-capacitor structures with the increasing MWCNT, resulting in a rapid increase in electrical conductivity and demonstrating a transition of the insulator to the semiconductor.

**Figure 12:**
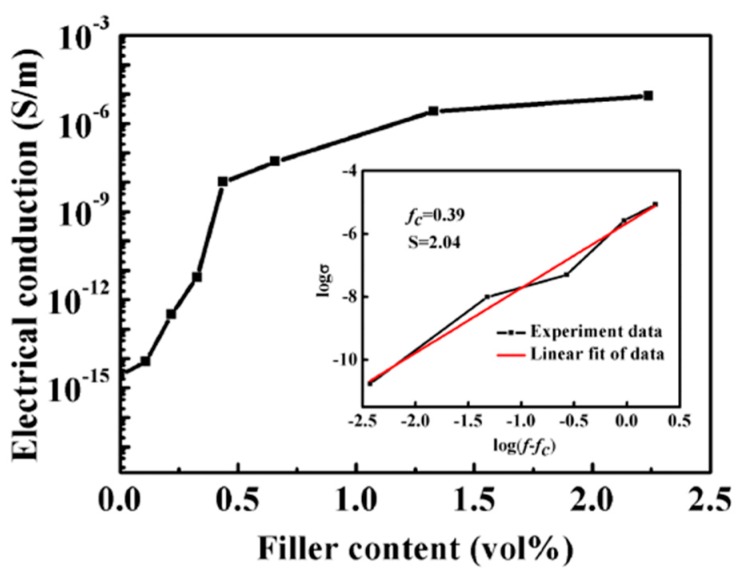
Electrical conductivity of MWCNT/PB-1 composite films as a function of filler content. Inset: the double-logarithmic plot of σ* versus *f − fc* with a least-squares fitting line to the experimental data of MWCNT/PB-1 composite films.

The conductivity of the MWCNT/PB-1 composite film as a function of filler volume fraction at 25 °C and 10^3^ Hz was shown in Figure 12. It should be mentioned that the content of the MWCNT was changed from the mass fraction (wt%) to the volume fraction (vol%). The pure PB-1 film had a very low AC conductivity and exhibited insulator properties. As the volume content of the MWCNT gradually increased, the electrical conductivity jumped sharply in the range of 0.15% to 0.5% by volume, indicating the transition of the insulator to the semiconductor. The electrical conductivity of the composite film depended primarily on the electrical conductivity of the conductor. According to the percolation threshold theory, the conductivity of the MWCNT/PB-1 composite film could be expressed by the general power law model. The equation is as follows [37]:

(3) $$\sigma^{*}=\sigma {\left(f-f_{c}\right)}^{s}$$

Where σ* denotes the electrical conductivity of the composite film. σ is the bulk conductivity of the filler. In Equation (3), *f* is the volume fraction of the filler, and s is the critical index describing the rapid change of the near-permeability threshold (*fc*). As shown in Figure 12, for the double logarithmic plot of conductivity and *f − fc*, the conductivity of the composite membrane met the percolation prevalence predicted by Equation (3). In particular, when *fc* = 0.391 vol%, and *s* = 2.04, the fitted line was very consistent with the experimental data.

#### 3.3.2. Dielectric Properties of MWCNT/PB-1 Films with Different Contents of MWCNT

**Figure 13:**
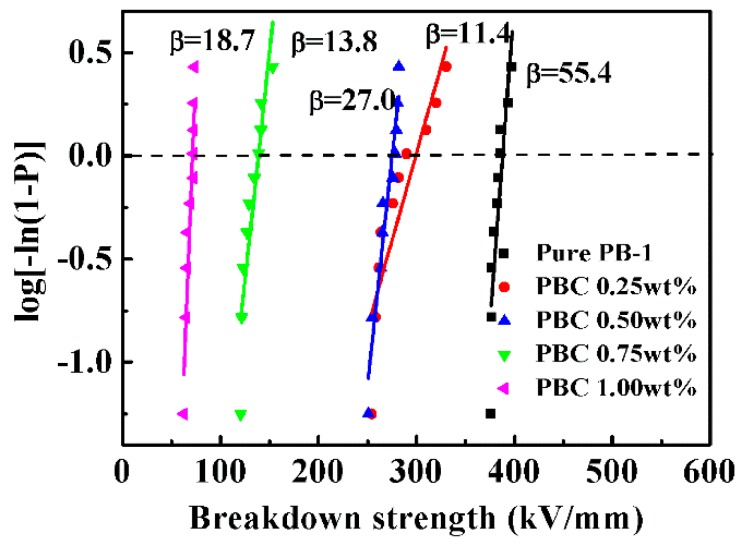
Dielectric breakdown strength of pure PB-1 film and the MWCNT/PB-1 composite films.

The effects of different contents of MWCNT on the puncture resistance of PB-1 composite films were investigated, as shown in Figure 13. According to the Weibull distribution curve of Figure 13, the breakdown strength of MWCNT/PB-1 composite films decreased sharply with the increase of MWCNT. Since the MWCNT were conductors, the percolation threshold point appeared near 1.0 wt% of the MWCNT addition. The film had a breakdown strength of at least 72.5 kV/mm. When the content of MWCNT exceeded 1.0 wt%, a large amount of micro-capacitor was formed, which was converted from an insulator to a semiconductor, consistent with SEM results. Ergo, the composite film was rapidly broken down under the action of an external electric field. It should be noted that when we tested the breakdown strength of the PB-1 composite film with MWCNT of 2.0 wt%, the leakage current was so large, that it led to the breakdown of the film at 2 kV/mm. Therefore, the maximum addition amount of the MWCNT was 1.0 wt% when the MWCNT/PB-1 composite films were studied as energy storage capacitors.

**Figure 14:**
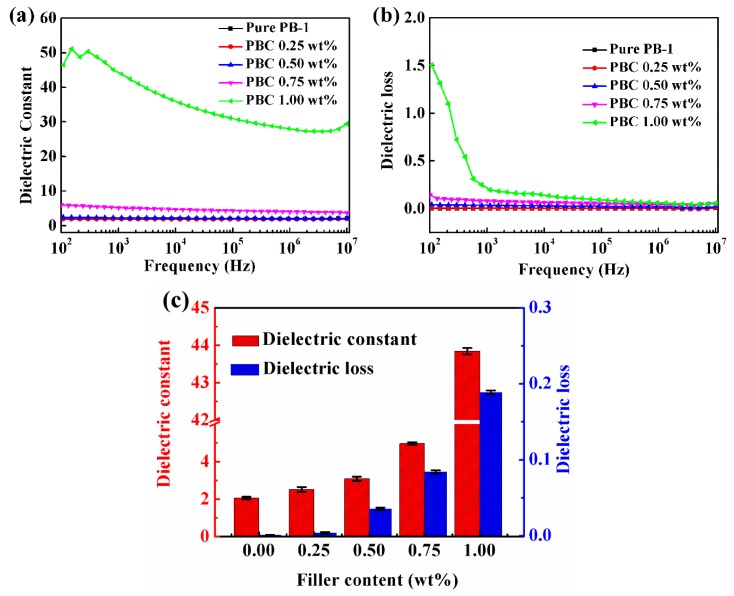
Frequency dependence of a dielectric constant (ε’) (**a**) and dielectric loss (tan δ) (**b**) of MWCNT/PB-1 composite films with different MWCNT contents; and (**c**) dielectric properties of MWCNT/PB-1 composite films at 10^3^ Hz.

Figure 14 showed the relationship between the ε’ and tan δ of the PB-1 composite film as a function of frequency. It could be seen from Figure 14a that the ε’ of the MWCNT/PB-1 composite films increased with the increase of MWCNT content. When the frequency was 1 kHz, the ε’ of the MWCNT/PB-1 composite film with the MWCNT content of 1.0 wt% could reach 43.87, which was 20 times that of pure PB-1 film. When the content of MWCNT was low, the dependence of the ε’ of the composite film on frequency was very low. From the perspective of polarization, the polarization followed the change of the electric field in the test frequency range. Therefore, its ε’ hardly changed with the change of frequency. When the mass fraction of MWCNT was 1.0 wt%, the ε’ of the film decreased rapidly with the increase of frequency. In the scanning interval of 10^2^–10^7^ Hz, the ε’ of the composite film decreased from 46.50 to 29.46. The reason for this was that the formation of the conductive network and the degree of interfacial polarization increased when the carbon nanotube content was close to the percolation threshold. When the frequency of the applied electric field changed, the polarized dipole lagged behind the electric field, so that the internal polarization of the material was not sufficient, resulting in a decrease in ε’.

Figure 14b showed the relationship between the tan δ of MWCNT/PB-1 composite films with different MWCNT contents and frequencies. It could be seen that the tan δ of the film increased with the increase of MWCNT content due to the increased structural loss and interfacial polarization loss caused by the internal defects and interface of the composite films. In addition, as the number of conductive particles increased, the electrical conductivity and the loss of the composite film increased. When the mass fraction of MWCNT was 1.0 wt%, which was close to the percolation threshold, the tan δ of the film increased remarkably due to the increased polarization loss generated by both the interface and the leakage current formed inside the composite film via the adding of MWCNT.

The relationship between ε’, tan δ, and the MWCNT content of MWCNT/PB-1 composite films at 25 °C and 10^3^ Hz are shown in Figure 14c. It can be seen that as the content of MWCNT increased, the ε’ and tan δ of the composite films increased remarkably. This phenomenon was attributed to the formation of micro-capacitor structures and the enhancement of Maxwell–Wagner–Sillars (MWS) interfacial polarization [38]. Under the action of an external electric field, the interface of the phase and the number of micro-capacitors formed per unit area increased with the increase of MWCNT content. When the content of MWCNT was low, the good dispersion in the matrix created excellent polarization potential. A large amount of free charge accumulated at the interface, so the ε’ increased with the increase of MWCNT content, and the dielectric loss caused by polarization increased accordingly. The changes in dielectric properties for the MWCNT/PB-1 composite films prepared by us conformed to the typical percolation threshold model [37]. In our work, it was appropriate to control the addition of MWCNT at 1.0 wt%, in which the prepared MWCNT/PB-1 composite film had a breakdown strength of 72.5 kV/mm, a high dielectric constant of 43.9 and a low loss of 0.19 at 25 °C under 10^3^ Hz. The dielectric properties of the MWCNT/PB-1 composite films in our work were much better for charge storage applications, compared with those previously reported in the literature, as shown in Table 2 [18,19,39,40,41].

## 4. Conclusions

Pure PB-1 films and MWCNT/PB-1 composite films with different phase transition times were prepared by a solution casting method. The crystallization properties of the film were studied. It was found that high temperature annealing caused the formation of crystal form I and crystal form II during solution crystallization. And as the phase transition time was prolonged, form II transformed into form I. The introduction of MWCNT changed the nucleation mode and accelerated the rate of phase transformation. When the additional amount of MWCNT was less than 1.0 wt%, the crystallization rate and crystallization degree of MWCNT/PB-1 composite films could be improved. The dielectric properties of the film were related to the crystal type and internal molecular structure. The phase transition time was prolonged, and the stable crystal form I content in the film was gradually increased. Furthermore, the dielectric constant and the breakdown field strength of the pure PB-1 films and the MWCNT/PB-1 composite films were improved, and the dielectric loss was lowered. It was also found that the introduction of MWCNT had a dual function of simultaneously improving the crystallization and dielectric properties of the PB-1 composite films. The percolation threshold of the MWCNT/PB-1 composite films was 0.39 vol% (close to 1.0 wt%). It should be noted that the dielectric constant of the MWCNT/PB-1 composite films with 1.0 wt% MWCNT reached 43.9 at 25 °C and 10^3^ Hz, which was 20 times that of the original film and, moreover, the dielectric loss was only 0.19. Thus, the MWCNT/PB-1 composite films provide an effective route for the application of PB-1 films in the field of energy storage.

## Figures and Tables

**Table 1:** Melting and crystallization parameters derived from the DSC measurements for the composite films with different MWCNT contents.

Samples	T_m_ (°C)	ΔH_m_ (J/g)	T_c_ (°C)	ΔH_c_ (J/g)	X_c_ (%)
Pure PB-1	129.54	71.09	85.11	40.39	56.7
PBC-0.25 wt%	128.86	73.49	85.40	38.58	58.8
PBC-0.50 wt%	132.22	77.79	86.00	39.10	62.3
PBC-0.75 wt%	132.73	89.32	88.57	38.03	71.8
PBC-1.00 wt%	132.70	89.31	80.59	36.62	71.9
PBC-2.00 wt%	129.28	85.53	79.02	40.29	69.6

**Table 2:** Dielectric properties of different carbon material filled modified polyolefin materials, at the same frequency, 10^3^ Hz.

Polymer	Filler	Filler Content	Experimental Method	ε’	tan δ	Ref
Polypropylene	CNFs	1.9 vol%	Melt hot pressing	8.7	0.16	[39]
	MWCNT	2.0 wt%	Melt hot pressing	28	0.005	[40]
Polyvinylidene fluoride	MWCNT	3.0 wt%	Electrospinning	16.1	0.025	[41]
poly (1-butene)	Graphene nanosheets	2.0 wt%	Melt hot pressing	33.9	1.5	[18]
	MWCNT	7.0 wt%	Melt hot pressing	70.0	–	[19]
	MWCNT	1.0 wt%	Solution casting	43.9	0.19	our work
